# Redox State of Glutathione and Cysteine in Plasma Following Acute Stroke

**DOI:** 10.3390/antiox15010117

**Published:** 2026-01-16

**Authors:** Christopher McGinley, Oyinkansola Adeyemi, Oluwafayokemi Oyolola, Byron D. Ford, Gregory D. Ford

**Affiliations:** 1 Department of Anatomy, Howard University College of Medicine, 520 W. Street NW, Washington, DC 20059, USA; christopher.mcginley@bison.howard.edu (C.M.);; 2 Department of Natural Sciences, Southern University at New Orleans, 6400 Press Drive, New Orleans, LA 70126, USA

**Keywords:** ischemic stroke, oxidative stress, glutathione, glutathione disulfide, cysteine, cystine, biomarkers, transient middle cerebral artery occlusion (tMCAO), reactive oxygen species (ROS), reperfusion injury

## Abstract

Ischemic stroke is a major cause of long-term disability and death, with oxidative stress contributing substantially to post-ischemic injury. Reperfusion restores oxygen supply but simultaneously increases reactive oxygen species (ROS), amplifying secondary neuronal damage. This study examined time-dependent changes in systemic thiol redox status following transient middle cerebral artery occlusion (tMCAO) in rats. Plasma concentrations of cysteine (CySH), cystine (CySS), glutathione (GSH), and glutathione disulfide (GSSG), along with corresponding CySS/CySH and GSSG/GSH ratios and redox potentials (Eh), were evaluated 24 and 48 h after occlusion. At 24 h, thiol concentrations and redox ratios showed no significant differences between sham and tMCAO groups. By 48 h, a marked oxidative shift emerged, characterized by reduced CySH, elevated GSSG, and significant increases in both CySS/CySH and GSSG/GSH ratios. Redox potentials also demonstrated substantial oxidation at this time point. These findings indicate that prolonged ischemia–reperfusion induces systemic oxidative stress, with plasma redox status serving as a sensitive indicator of reperfusion-related injury. The results underscore the plasma redox status as a potentially sensitive biomarker of reperfusion-induced oxidative injury and support the therapeutic value of targeting redox imbalance to mitigate oxidative damage following stroke.

## 1. Introduction

Stroke, a leading cause of neurological disability, results from the interruption of cerebral blood flow, leading to ischemia and subsequent reperfusion injury [1,2]. During ischemia, diminished oxygen and glucose delivery trigger a cascade of biochemical disruptions beginning with rapid energy failure. ATP depletion impairs ion pumps, resulting in membrane depolarization and excessive glutamate release. This excitotoxic surge activates NMDA and AMPA receptors, causing uncontrolled calcium influx into neurons. Elevated intracellular calcium drives activation of phospholipases, proteases, endonucleases, and nitric oxide synthase, each of which contributes to the early overproduction of reactive oxygen species (ROS). These events occur concurrently with mitochondrial dysfunction, including collapse of membrane potential and impaired electron transport, which enhances superoxide formation and primes the tissue for oxidative damage [3,4].

The onset of reperfusion, although essential for restoring metabolic function, further amplifies ROS generation [1,2,3]. The sudden reintroduction of oxygen leads to accelerated mitochondrial electron leakage and increases the activity of enzymes such as xanthine oxidase and NADPH oxidase in neurons, glia, endothelial cells, and infiltrating immune cells. Neutrophil recruitment during the early reperfusion period fuels additional oxidative bursts through myeloperoxidase-dependent pathways. This heightened oxidative load overwhelms intrinsic antioxidant defenses, including glutathione (GSH), thioredoxin, superoxide dismutase, catalase, and peroxiredoxins, resulting in the oxidation of thiol-containing molecules and disruption of cellular redox homeostasis.

GSH is a tripeptide composed of three amino acids: cysteine, glycine, and glutamate. It functions as a potent antioxidant by neutralizing ROS and protecting cells from oxidative damage [5,6,7,8]. GSH also participates in cellular detoxification processes. GSH depletion reduces the ability of cells to scavenge and neutralize GSH serves as the principal intracellular redox buffer, maintaining redox homeostasis and protecting neurons from oxidative insult. GSH and its oxidized form, glutathione disulfide (GSSG), play a crucial role in maintaining redox homeostasis and cellular defense against oxidative stress. The GSH/GSSG ratio is a critical indicator of cellular redox status, with lower ratios reflecting oxidative stress and diminished antioxidant capacity. The ratio of reduced GSH to GSSG serves as an indicator of the redox state within cells. In a healthy state, GSH is present in higher concentrations compared to GSSG, resulting in a high GSH/GSSG ratio.

Cysteine redox refers to the reversible interconversion of cysteine between its oxidized form, cystine (CySS), and its reduced form, cysteine (CySH) [9]. In the context of oxidative stress, cysteine redox is highly relevant because cysteine is a derivative such as GSH, serves as a critical antioxidant and regulator of cellular redox balance [8,9,10]. This, in turn, exacerbates oxidative damage and amplifies the injury to brain cells. During normal cellular conditions, the ratio of CySH to CySS is relatively high, indicating a more reduced state within the cell. This high CySH/CySS ratio is important for maintaining the antioxidant capacity of the cell and protecting against oxidative damage on cellular components. However, under conditions of increased oxidative stress, the balance between CySH and CySS can be disrupted. This shift towards a more oxidized state is indicative of higher oxidative stress and compromised antioxidant defenses within the cell [9]. Recent studies implicate disturbances in GSH and CySH redox states as mediators of neuronal death [11,12,13].

Multiple interconnected pathogenic mechanisms converge to exacerbate oxidative injury in the ischemic brain [9,11,12,13]. Oxidative stress contributes to apoptotic and necrotic cell death by releasing pro-apoptotic factors and impairing respiratory capacity. Endothelial dysfunction and blood–brain barrier (BBB) breakdown expose neural tissue to blood-derived cytokines, proteases, and iron, further intensifying oxidative stress. Simultaneously, microglial activation and astrocytic dysfunction propagate inflammatory signaling and ROS production, while impairing nitric oxide bioavailability and vascular reactivity. These inflammatory and vascular changes further shift extracellular cysteine/cystine (CySH/CySS) and intracellular GSH/GSSG redox couples toward oxidation, reinforcing the oxidative environment across both local and systemic compartments.

Collectively, these etiopathogenetic processes illustrate how oxidative stress evolves from both ischemia-induced metabolic collapse and reperfusion-driven biochemical acceleration. The delicate balance between ROS generation and antioxidant defense becomes profoundly disrupted, establishing oxidative stress as a central driver of acute and subacute neuronal injury. Understanding how these mechanisms are reflected in systemic redox biomarkers, such as plasma GSH/GSSG and CySH/CySS ratios, may provide important insights into the progression of post-stroke pathology and identify therapeutic targets aimed at restoring redox equilibrium. This study investigates the temporal dynamics of these redox couples following transient middle cerebral artery occlusion (tMCAO) in rats, with the goal of determining whether plasma redox status reflects the evolving oxidative burden during early and delayed phases of reperfusion injury.

## 2. Materials and Methods

### 2.1. Animals

All surgical procedures were performed by sterile/aseptic techniques in accordance with institutional guidelines. All procedures were approved by the Institutional Animal Care and Use Committees at Morehouse School of Medicine (Protocol #A-20160020) and the University of California–Riverside (Protocol #20190021) and were conducted in accordance with the ARRIVE guidelines and the AVMA Guidelines for the Euthanasia of Animals. Male adult Sprague–Dawley rats (*Rattus norvegicus*, 250–300 g; Charles River Laboratory International, Inc., Wilmington, MA, USA) were housed in standard cages in a temperature-controlled room (22 ± 2 °C) on a 12 h reverse light–dark cycle with ad libitum access to food and water. Animals were anesthetized with a ketamine/xylazine solution (10 mg/kg, i.p.) and left middle cerebral artery occlusion (MCAO) was induced by the intraluminal suture method, as previously described [14]. Briefly, the left common carotid artery (CCA) was exposed and dissected free from surrounding nerves and fascia. The occipital artery and superior thyroid artery branches of the ECA were coagulated and dissected distally. The internal carotid artery (ICA) was isolated and separated from the adjacent vagus nerve. Finally, the pterygopalatine artery was ligated close to its origin with a 6-0 silk suture. A 40 mm 3-0 nylon monofilament (Harvard Apparatus, Holliston, MA, USA), coated with poly-L-lysine and rounded at the tip by brief heating, was introduced via the ECA and advanced into the ICA to the Circle of Willis to occlude the origin of the left middle cerebral artery. The filament was advanced 18–20 mm beyond the CCA bifurcation. After 1.5 h of ischemia, the filament was withdrawn to allow reperfusion. Animals were allowed to recover and were reperfused for 24 or 48 h before sacrifice. Cerebral blood flow was continuously monitored during occlusion and reperfusion using laser Doppler flowmetry (Perimed, Ardmore, PA, USA). The probe was positioned 7 mm lateral and 2 mm posterior to the bregma through a thinned cranial window to confirm successful arterial occlusion and reperfusion. A ≥70% reduction in CBF after occlusion was required for inclusion; all animals survived surgery, and no animals were excluded. Core body temperature was monitored with a rectal probe and maintained at 37.8 °C with a Homeothermic Blanket Control Unit (Harvard Apparatus) during anesthesia. Animals were sacrificed 24 (*n* = 3) and 48 h (*n* = 14) after tMCAO; Sham animals were sacrificed 3 h after surgery 24 (*n* = 5) and 48 (*n* = 6). Euthanasia was performed by overdose with Euthanasia-B (90 mg/kg injected IP), in compliance with AVMA guidelines.

### 2.2. Immunohistochemistry

Infarct volumes were assessed using 2,3,5-triphenyltetrazolium chloride (TTC) staining, as previously described [15]. Animals were sacrificed Sham 24 (*n* = 3), Sham 48 (*n* = 5), tMCAO 24 (*n* = 4), and tMCAO 48 (*n* = 4)], after surgery. Animals were deeply anesthetized and transcardially perfused with ice-cold saline. Brains were rapidly removed, placed in a chilled coronal matrix, and sectioned into 2 mm thick slices. Slices were incubated in 2% TTC (*w*/*v* in PBS) at 37 °C for 15–20 min in the dark, with gentle agitation to ensure uniform staining. Viable tissue reduced TTC to a deep red formazan product, whereas infarcted areas remained pale. Following staining, slices were rinsed in PBS and post-fixed in 4% paraformaldehyde overnight at 4 °C. Digital images were captured under consistent lighting conditions, and infarct area was quantified using ImageJ (v1.54). Infarct volume was calculated as infarct area × slice thickness and summed across slices; edema-corrected infarct volume was derived by subtracting non-infarcted ipsilateral volume from the contralateral hemisphere volume.

Neurodegeneration was visualized using Fluoro-Jade B (FJB) staining, *n* = 3 for each group, as previously described [16]. Mounted tissue sections were sequentially immersed in 1% NaOH in 80% ethanol for 5 min, 70% ethanol for 2 min, and distilled water for 2 min, followed by incubation in 0.06% potassium permanganate for 10 min to reduce background fluorescence. Sections were then stained in 0.0004% FJB prepared in 0.1% acetic acid for 20–30 min in the dark, rinsed in distilled water, and air-dried. Once dry, sections were cleared briefly in xylene and cover-slipped with a fluorescence-compatible mounting medium. Stained sections were imaged using an epifluorescence microscope with GFP filter settings for FJB-positive degenerating neurons.

### 2.3. Mass Spectrometry Analysis of Reduced/Oxidized Thiols

Samples processed according to manufacturer standard protocols [17] and peptides sequences were determined by tandem mass spectrometry (LC/MS/MS), using an LTQ Ion Trap Mass Spectrometer by Thermo Fischer Scientific (Waltham, MA, USA). Standard protocol included precipitation followed by trypsinization. The peptides were then reduced in DTT (10 mmol/L) and alkylated with 15 mM iodoacetic acid. Finally, samples were digested on spectrometry grade trypsin and acidified in 0.1% formic acid. Protein sequence identification and analysis were further performed using Xcalibur software (version 2.2; Thermo Fisher Scientific, Waltham, MA, USA) ProteoIQ software (version 2.3.08; NuSep, Inc., Bogart, GA, USA), and homologous human proteins were identified using BLASTp (v2.16.0) through the National Center for Biotechnology Information.

### 2.4. Calculated Redox Potentials (Eh) for the CySS/CySH and GSSG/GSH

Redox potentials (Eh) for the cysteine/cystine and glutathione/glutathione disulfide redox couples were calculated according to the Nernst equation:

$$E_{h}=E{}^{\circ}+\frac{RT}{nF}ln\frac{\left[Ox\right]}{[Red{]}^{2}}$$

where E° is the standard potential of the redox couple, R is the gas constant (8.314 J·K^−1^·mol^−1^), T is the absolute temperature (298 K), *n* is the number of electrons transferred (*n* = 2), and F is the Faraday constant (96,485 C·mol^−1^).

For biological systems at 25 °C, this simplifies to:

$$E_{h}=E{}^{\circ}+30{log}_{10}(\frac{\left[Ox\right]}{[Red{]}^{2}})$$

The standard potentials used were −250 mV for the cysteine/cystine (CySS/CySH) couple and −264 mV for the glutathione (GSSG/GSH) couple.

Accordingly, the specific equations applied were:

$$E_{h(CySS/CySH)}=-250+30{log}_{10}(\frac{\left[CySS\right]}{[CySH{]}^{2}})$$

$$E_{h(GSSG/GSH)}=-264+30{log}_{10}(\frac{\left[GSSG\right]}{[GSH{]}^{2}})$$

### 2.5. Statistical Analysis

Data were originally tabulated in Microsoft Excel with the XLMiner (v4.0) Analysis Toolkit and imported into Python (v3.11) for quantitative analysis. All data were derived from experimentally measured plasma thiol and disulfide concentrations (CySS, CySH, GSH, GSSG) collected from sham-operated and MCAO rats at 24 h and 48 h post-occlusion. Data computation of redox ratios, redox potentials (Eh) calculated using the Nernst equation. Statistical testing was performed using the following libraries: pandas (v2.2.2) for data cleaning and organization, numpy (v1.12.4) for ratio and redox potential calculations, and scipy.stats (1.13.1) for *t*-tests and *p*-value computation. Figures were generated using matplotlib (v3.9.2), seaborn (v0.13.2), and Microsoft Excel. All summary statistics (mean ± standard deviation) were preformed using the Mann–Whitney U test as a non-parametric test to verify distribution, and the Welch’s *t*-tests, to determine variance, conducted in Python. The minimum level of significance was set at *p* < 0.05.

## 3. Results

### 3.1. Assessment of Cerebral Blood Flow and Infarct Formation Following Transient Middle Cerebral Artery Occlusion

**Figure 1:**
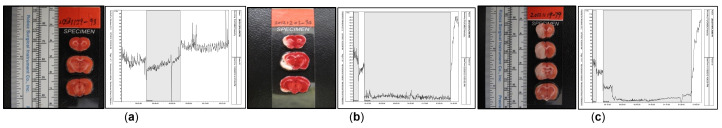
Triphenyltetrazolium chloride (TTC) staining and real-time cerebral blood flow (CBF) recordings in sham and transient middle cerebral artery occlusion (tMCAO) rats. (**a**) rat brain sections following sham 24 h (*n* = 3) and sham 48 h (*n* = 5) surgery, (TTC) staining shows uniformly stained brain slices with no infarct areas, indicating preserved tissue viability. The corresponding CBF trace demonstrates stable cerebral perfusion throughout the recording period, confirming the absence of ischemic insult. (**b**) rat brain sections following tMCAO, 24 h (*n* = 4), TTC staining reveals distinct unstained regions in the ipsilateral hemisphere, consistent with infarcted tissue. Real-time CBF monitoring shows an overall reduction of approximately 17.6% compared to baseline levels. (**c**) rat brain sections following tMCAO, 48 h (*n* = 4), TTC staining demonstrates more extensive infarction, evident by larger unstained areas in the affected hemisphere. The CBF recording shows an overall reduction of 18.5% relative to baseline. These data collectively illustrate the relationship between reduced CBF during tMCAO, and the corresponding extent of cerebral infarction visualized by TTC staining.

To evaluate the effects of transient middle cerebral artery occlusion (tMCAO) on cerebral blood flow (CBF) and ischemic injury, real-time CBF measurements were recorded during and after occlusion, and infarct size was assessed using 2,3,5-triphenyltetrazolium chloride (TTC) staining (Figure 1). Three representative experimental groups were analyzed: sham-operated animals, rats subjected to 24 h of tMCAO, and rats subjected to 48 h of tMCAO.

In sham-operated rats, CBF remained stable throughout the recording period, showing no significant fluctuations during the surgical procedure (Figure 1a). TTC staining revealed uniform deep red coloration across all brain slices, indicating intact mitochondrial function and the absence of ischemic infarction. These findings confirm that the surgical manipulation alone did not affect cerebral perfusion or tissue viability.

In contrast, rats subjected to 24 h of tMCAO exhibited a rapid and sustained reduction in CBF immediately following the occlusion event, with an average reduction of 17.6% from baseline levels (Figure 1b). TTC staining of the corresponding brain sections showed distinct pale regions in the ipsilateral cortex and striatum, consistent with infarcted tissue. These findings confirm the successful induction of focal ischemia and the subsequent development of infarction following transient MCA occlusion.

Similarly, rats exposed to 48 h of tMCAO demonstrated a comparable reduction in CBF, with an average decrease of 18.5% relative to baseline (Figure 1c). The prolonged duration of ischemia was associated with a larger infarct area on TTC staining compared to the 24 h group, characterized by extensive unstained regions encompassing the cortical and subcortical territories. This suggests that prolonged ischemic duration exacerbates neuronal damage and infarct expansion.

Together, these results indicate that tMCAO reliably produces a marked reduction in cerebral perfusion, leading to reproducible ischemic injury that can be visualized by TTC staining. The extent of infarction correlates with both the duration and magnitude of CBF reduction.

### 3.2. Fluoro-Jade B Staining Reveals Neuronal Degeneration 48 h After Transient Middle Cerebral Artery Occlusion

**Figure 2:**
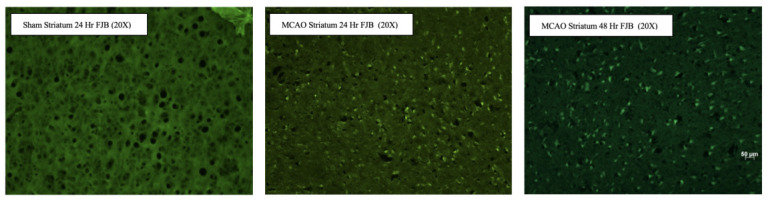
Fluoro-Jade B staining reveals increased striatal neurodegeneration following MCAO. Representative FJB–stained images of the striatum at 20× magnification. Sham-operated animals at 24 h show minimal FJB-positive staining, indicating limited neuronal degeneration. In contrast, MCAO animals display increased FJB fluorescence at 24 h, consistent with acute ischemia-induced neurodegeneration. Elevated FJB staining persists at 48 h post-MCAO, suggesting sustained neuronal injury in the striatum. Green fluorescence denotes FJB-positive degenerating neurons.

In sham-operated animals at 24 h, minimal FJB fluorescence was observed (Figure 2), indicating a low level of neuronal degeneration and preservation of striatal tissue integrity. In contrast, animals subjected to MCAO exhibited a marked increase in FJB-positive staining within the striatum at 24 h post-injury. Numerous brightly fluorescent cells were evident, suggesting acute neuronal degeneration following ischemic insult. The distribution of FJB-positive cells was widespread throughout the striatal parenchyma, reflecting substantial vulnerability of this region to cerebral ischemia. The distribution and density of FJB-positive cells confirmed widespread neuronal injury in the ischemic hemisphere 48 h post-tMCAO, consistent with robust neurodegeneration during the subacute phase of ischemic injury. These findings validate the presence of extensive neurodegeneration in the ischemic territory following tMCAO and further corroborate the histopathological and physiological evidence of cerebral infarction obtained from TTC and CBF analyses.

### 3.3. Plasma Thiol and Disulfide Concentrations

Quantitative analysis of plasma thiol and disulfide concentrations and ratios revealed time-dependent changes following tMCAO (Table 1). At 24 h post-occlusion, no significant differences (*p* > 0.05) were observed between sham and tMCAO animals in the concentrations of CySS, CySH, GSH, or GSSG. CySS and CySH levels remained within physiological ranges (25–27 µM and 14–15 µM, respectively) [18], and the GSH and GSSG concentrations showed no measurable oxidation, indicating preserved systemic redox balance during the early reperfusion phase.

**Table 1:** Plasma thiol and disulfide concentrations in sham and MCAO rats at 24 and 48 h post-occlusion. Plasma concentrations of cystine (CySS), cysteine (CySH), reduced glutathione (GSH), and oxidized glutathione (GSSG) were measured in sham-operated and transient middle cerebral artery occlusion (MCAO) rats at 24 and 48 h following reperfusion. Data are expressed as mean ± SD (µM). Statistical comparisons between sham and MCAO groups at each time point were performed using Welch’s *t*-test. Significant differences (*p* < 0.05). (* = *p* ≤ 0.05, ** = *p* ≤ 0.01).

Plasma Thiol Concentrations					
Parameter	Time	Sham (Mean ± SD)	MCAO (Mean ± SD)	*p*-Value	Direction of Change
CySS (µM)	24 h	25.50 ± 3.76	27.47 ± 2.44	0.405	—
CySH (µM)	24 h	15.24 ± 2.25	14.40 ± 4.42	0.782	—
CySS (µM)	48 h	26.55 ± 4.09	25.45 ± 3.08	0.602	—
CySH (µM)	48 h	15.31 ± 1.86	12.22 ± 2.38	0.016 *	Decreased in MCAO
GSH (µM)	24 h	3.39 ± 0.71	2.83 ± 1.16	0.498	—
GSSG (µM)	24 h	0.22 ± 0.06	0.16 ± 0.01	0.08	—
GSH (µM)	48 h	2.67 ± 0.57	2.67 ± 0.73	0.995	—
GSSG (µM)	48 h	0.11 ± 0.05	0.22 ± 0.09	0.006 **	Increased in MCAO

**Figure 3:**
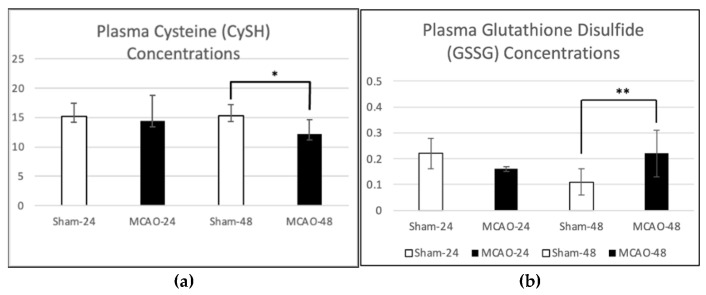
Plasma cysteine (CySH) and glutathione disulfide (GSSG) concentrations following transient middle cerebral artery occlusion (MCAO). (**a**) Plasma levels of reduced cysteine (CySH) were measured in sham-operated (sham 24 h, *n* = 5 and sham 48 h, *n* = 6) and tMCAO (tMCAO 24 h, *n* = 3 and tMCAO (*n* = 14) rats post-occlusion. Data are expressed as mean ± SD (µM). using the Mann–Whitney U test and Welch’s *t*-test no significant difference was observed between sham and MCAO groups at 24 h (*p* = 0.782). By 48 h, CySH concentrations were significantly decreased in the MCAO group compared with sham (*p* = 0.016), indicating depletion of extracellular reducing equivalents during reperfusion-associated oxidative stress. (**b**) Plasma concentrations of oxidized glutathione (GSSG) were measured in sham-operated and MCAO rats at 24 and 48 h post-occlusion. Data are presented as mean ± SD (µM). No significant difference was observed between sham and MCAO groups at 24 h (*p* = 0.080). At 48 h, GSSG levels were significantly elevated in the MCAO group compared with sham (*p* = 0.006), indicating enhanced oxidation of the glutathione pool and impaired redox recovery during reperfusion. (* = *p* ≤ 0.05, ** = *p* ≤ 0.01).

By 48 h, however, plasma concentrations reflected a clear oxidative shift in the tMCAO group. Reduced CySH levels were significantly decreased compared with time-matched shams (12.22 ± 2.38 vs. 15.31 ± 1.86 µM, *p* = 0.016) (Figure 3a), while GSSG concentrations were significantly elevated (0.22 ± 0.09 vs. 0.11 ± 0.05 µM, *p* = 0.006) (Figure 3b). These findings indicate progressive depletion of plasma reducing equivalents and accumulation of oxidized thiols during late reperfusion.

### 3.4. Plasma Redox Ratios

**Figure 4:**
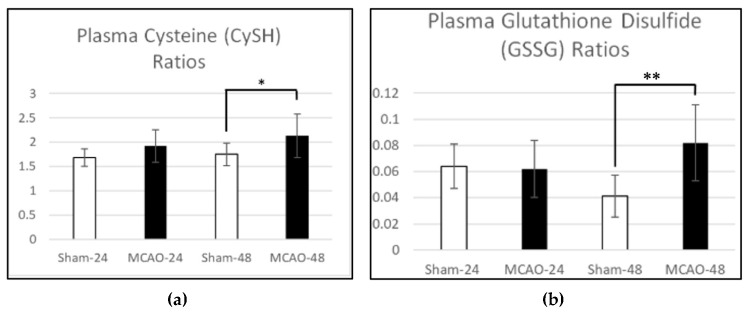
Plasma cysteine (CySH) glutathione disulfide (GSSG/GSH) ratios following transient middle cerebral artery occlusion (MCAO). (**a**) Plasma cysteine redox ratios (CySS/CySH) were measured in sham-operated and MCAO rats at 24 and 48 h post-occlusion. Data are presented as mean ± SD. No significant differences were observed between sham and MCAO animals at 24 h (*p* = 0.425). By 48 h, the CySS/CySH ratio was significantly increased in the MCAO group compared with sham (*p* = 0.031), indicating oxidation of the extracellular thiol pool and a shift toward a more oxidized plasma redox state. The asterisk (*) denotes *p* < 0.05. (**b**) Plasma GSSG/GSH ratios were calculated in sham-operated and MCAO rats at 24 and 48 h post-occlusion. Data are shown as mean ± SD. No significant difference was detected between groups at 24 h (*p* = 0.862). By 48 h, the GSSG/GSH ratio was significantly elevated in the MCAO group compared with sham (*p* = 0.002), indicating oxidation of the intracellular glutathione pool and loss of reducing capacity during reperfusion injury. (* = *p* ≤ 0.05, ** = *p* ≤ 0.01).

The alterations in individual thiol concentrations were accompanied by significant increases in plasma redox ratios, using the Mann–Whitney U test and Welch’s *t*-test (Table 2). At 24 h, both CySS/CySH and GSSG/GSH ratios were similar between sham and tMCAO animals (*p* = 0.425 and *p* = 0.862, respectively). By 48 h, however, the CySS/CySH ratio increased from 1.75 ± 0.23 in shams to 2.13 ± 0.45 in tMCAO rats (*p* = 0.031) (Figure 4a), while the GSSG/GSH ratio rose from 0.041 ± 0.016 to 0.082 ± 0.029 (*p* = 0.002) (Figure 4b). These ratio changes signify a pronounced oxidation of both extracellular and intracellular thiol pools in plasma, corresponding to the biochemical transition from a reduced to an oxidized systemic environment.

**Table 2:** Plasma thiol redox ratios in sham-operated and MCAO rats at 24 and 48 h post-occlusion. Plasma redox ratios for the cysteine/cystine (CySS/CySH) and glutathione disulfide/glutathione (GSSG/GSH) couples were calculated in sham-operated and transient middle cerebral artery occlusion (MCAO) rats at 24 and 48 h post-occlusion. Data are expressed as mean ± SD. Statistical comparisons were performed using Welch’s *t*-test, with significance defined as *p* < 0.05. At 24 h, no significant differences in redox ratios were observed between groups. By 48 h, both CySS/CySH and GSSG/GSH ratios were significantly elevated in the MCAO group compared with sham controls (*p* = 0.031 and *p* = 0.002, respectively), indicating oxidation of both extracellular and intracellular thiol redox systems. (* = *p* ≤ 0.05).

Plasma Thiol Redox Ratios					
Parameter	Time	Sham (Mean ± SD)	MCAO (Mean ± SD)	*p* -Value	Change
CySS/CySH Ratio	24 h	1.68 ± 0.18	1.92 ± 0.33	0.425	—
GSSG/GSH Ratio	24 h	0.064 ± 0.017	0.062 ± 0.022	0.862	—
CySS/CySH Ratio	48 h	1.75 ± 0.23	2.13 ± 0.45	0.031 *	Oxidized
GSSG/GSH Ratio	48 h	0.041 ± 0.016	0.082 ± 0.029	0.002 *	Oxidized

Together, the concentration and ratio data demonstrate that systemic redox homeostasis remains intact at 24 h post-occlusion but becomes significantly oxidized by 48 h, highlighting the delayed onset of plasma oxidative stress following cerebral ischemia and reperfusion.

### 3.5. Plasma Redox Potentials

Redox potentials (Eh) for the CySS/CySH and GSSG/GSH couples were calculated for each sample using the Nernst equation to assess the oxidative status of extracellular and intracellular thiol systems following tMCAO (Table 3). The resulting data were summarized to assess directional changes in redox balance (Table 4). At 24 h post-occlusion, no significant differences in redox potential were observed between sham and tMCAO group for either redox couple. The mean Eh (CySS/CySH) was −278.7 ± 2.8 mV in sham and −275.5 ± 8.2 mV in MCAO animals (*p* = 0.574), while the mean Eh (GSSG/GSH) values were −316.0 ± 4.6 mV and −313.7 ± 10.3 mV, respectively (*p* = 0.744). These findings indicate that plasma redox balance remained largely stable within the first 24 h of reperfusion.

By 48 h, a significant oxidation of both redox couples was evident in the tMCAO group (Table 4). The Eh (CySS/CySH) became more positive (oxidized) in tMCAO animals compared with time-matched shams (−272.7 ± 4.9 vs. −278.4 ± 2.8 mV; *p* = 0.008), and a similarly, the Eh (GSSG/GSH) became more positive (oxidized) in the MCAO animals compared to time-matched shams (−309.5 ± 5.7 vs. −319.1 ± 6.1 mV; *p* = 0.019). The magnitude of the redox shift (5–10 mV) indicates a substantial oxidation of both extracellular and intracellular thiol-disulfide systems during late reperfusion.

Collectively, these data demonstrate that plasma redox potentials remain stable during early reperfusion but undergo a significant oxidative shift by 48 h after transient cerebral ischemia, reflecting progressive systemic oxidation and impaired redox buffering capacity.

**Table 3:** Plasma redox potentials (Eh) for cysteine/cystine (CySS/CySH) and glutathione disulfide/ glutathione (GSSG/GSH) across experimental groups. Redox potentials (Eh) were calculated using the Nernst equation (Eh = E° + 30 log([CySS]/[CySH]^2^), E° = −250 mV) for plasma cysteine/cystine couples and (Eh = E° + 30 log([GSSG]/[GSH]^2^), E° = –264 mV) in sham-operated and transient middle cerebral artery occlusion (MCAO) rats at 24 h and 48 h post-occlusion. Data are presented as mean ± SD (mV).

Calculation of REDOX Potential								
	Concentration, μM		30 × (log (CySS/CySH^2^)	Eh CySS, mV (Eo = −250)	Concentration, μM		30 × (log (GSSG/GSH ^2^)	Eh GSSG, mV (Eo = −264)
	CySS	CySH			GSH	GSSG		
Sham 24	19.22	11.83	154.1	**−95.9**	2.99	0.240	132.9	**−131.1**
Sham 24	27.49	14.33	153.8	**−96.2**	3.08	0.224	131.2	**−132.8**
Sham 24	28.70	16.45	150.8	**−99.2**	3.58	0.273	129.9	**−134.1**
Sham 24	24.98	17.64	147.1	**−102.9**	4.21	0.223	123	**−141**
Sham 24	27.10	15.95	150.8	**−99.2**	3.10	0.123	123.2	**−140.8**
MCAO 24	26.53	18.37	146.9	**−103.1**	4.07	0.160	119.5	**−144.5**
MCAO 24	30.24	15.19	153.5	**−96.5**	2.66	0.169	131.3	**−132.7**
MCAO 24	25.63	9.64	163.2	**−86.8**	1.77	0.146	140.1	**−123.9**
Sham 48	29.74	16.55	151.1	**−98.9**	3.15	0.180	127.8	**−136.2**
Sham 48	31.38	14.71	154.8	**−95.2**	2.12	0.121	132.9	**−131.1**
Sham 48	25.51	17.78	147.2	**−102.8**	3.41	0.129	121.4	**−142.6**
Sham 48	21.07	13.04	152.8	**−97.2**	2.31	0.081	125.4	**−138.6**
Sham 48	25.05	14.47	152.3	**−97.7**	2.37	0.044	116.8	**−147.2**
Sham 48	25.26	12.90	155.4	**−94.6**	2.58	0.180	133	**−131**
MCAO 48	25.16	10.74	160.2	**−89.8**	2.97	0.368	138.6	**−125.4**
MCAO 48	30.92	13.83	156.3	**−93.7**	3.11	0.252	132.5	**−131.5**
MCAO 48	26.27	15.10	151.8	**−98.2**	3.46	0.359	134.3	**−129.7**
MCAO 48	26.74	9.66	163.7	**−86.3**	2.75	0.207	133.1	**−130.9**
MCAO 48	29.93	16.57	151.1	**−98.9**	4.01	0.291	127.7	**−136.3**
MCAO 48	26.30	11.99	157.9	**−92.1**	3.35	0.222	128.9	**−135.1**
MCAO 48	23.73	12.60	155.2	**−94.8**	2.93	0.251	134	**−130**
MCAO 48	28.37	9.81	164.1	**−85.9**	2.13	0.337	146.1	**−117.9**
MCAO 48	24.86	13.81	153.5	**−96.5**	2.99	0.168	128.2	**−135.8**
MCAO 48	26.40	8.22	167.8	**−82.2**	1.46	0.154	145.8	**−118.2**
MCAO 48	22.92	12.53	154.9	**−95.1**	1.79	0.133	138.5	**−125.5**
MCAO 48	19.05	9.49	159.8	**−90.2**	1.45	0.072	136	**−128**
MCAO 48	24.71	14.92	151.4	**−98.6**	2.69	0.156	130	**−134**
MCAO 48	21.10	11.20	156.8	**−93.2**	2.44	0.137	130.9	**−133.1**

**Table 4:** Plasma thiol redox potentials (Eh) in sham and MCAO rats at 24 and 48 h post-occlusion. At 24 h, no significant changes in Eh were observed between groups, indicating maintained redox equilibrium. By 48 h, however, both CySS/CySH and GSSG/GSH couples exhibited significantly more positive (oxidized) Eh values in MCAO animals compared to sham controls (*p* = 0.008 and *p* = 0.019, respectively), reflecting systemic and intracellular oxidative stress induced by ischemia–reperfusion injury. (* = *p* ≤ 0.05, ** = *p* ≤ 0.01).

Plasm Thiol Redox Potentials (Eh)						
Parameter	Time	Sham (mV ± SD)	MCAO (mV ± SD)	*p*-Value	Change	Interpretation
Eh (CySS/CySH)	24 h	−99.9 ± 2.9	−95.5 ± 7.8	0.574	—	No significant oxidation
Eh (CySS/CySH)	48 h	−97.7 ± 4.0	−92.6 ± 6.3	0.008 **	↑ Oxidized	Significant extracellular oxidation
Eh (GSSG/GSH)	24 h	−136.0 ± 5.6	−133.7 ± 8.4	0.744	—	No significant change
Eh (GSSG/GSH)	48 h	−137.8 ± 7.0	−129.4 ± 7.4	0.019 *	↑ Oxidized	Significant intracellular oxidation

## 4. Discussion

The present study demonstrates that tMCAO produces a reproducible and time-dependent progression of ischemic injury, accompanied by a delayed but significant shift in systemic redox balance. Consistent reductions in CBF and the development of infarction confirmed the reliability of the MCAO model, while FJB staining verified extensive neurodegeneration within the ipsilateral cortex and striatum. Together, the physiological, histological, and biochemical data support the conclusion that redox imbalance emerges not during the early reperfusion phase but during the subacute period, reflecting secondary injury mechanisms initiated after the ischemic event.

A key finding of this study is that systemic redox homeostasis remains largely preserved at 24 h following tMCAO but shows a marked oxidative shift by 48 h. This pattern was evident across all measured thiol indices, including declining CySH levels, increased GSSG accumulation, elevated CySS/CySH and GSSG/GSH ratios, and a consistent shift toward more positive (oxidized) redox potentials in both cysteine and glutathione couples.

The delayed onset of oxidative imbalance aligns with the established biphasic nature of post-ischemic injury. Initial ischemic depolarization and excitotoxicity generate ROS acutely, but the most extensive oxidative damage often manifests later, driven by mitochondrial dysfunction, sustained inflammatory activation, BBB disruption, and immune cell infiltration. The 48 h time point coincides with peak microglial activation, increased NADPH oxidase activity, and ongoing reperfusion-related ROS production. The factors collectively exhaust antioxidant reserves, overwhelm glutathione recycling systems, and promote systemic oxidation.

Cysteine and glutathione redox systems play central roles in maintaining cellular and extracellular redox balance [18,19,20]. The significant reduction in plasma CySH at 48 h suggests impaired extracellular antioxidant buffering or increased conversion to CySS in response to rising oxidative burden. Elevated plasma GSSG reflects saturation of glutathione reductase activity and insufficient regeneration of GSH, a hallmark of oxidative stress in ischemia–reperfusion injury.

Because the CySS/CySH couple predominantly reflects plasma and vascular redox status, its oxidation may have functional consequences, including impaired endothelial nitric oxide signaling, increased protein S-glutathionylation, and heightened vascular inflammation [18,20]. In contrast, the GSSG/GSH ratio, though measured in plasma, is considered a sensitive surrogate for intracellular redox changes in circulating immune cells and erythrocytes [17]. Thus, the concurrent oxidation of both systems suggests a coordinated systemic response to cerebral ischemia, extending beyond local brain tissue injury.

**Figure 5:**
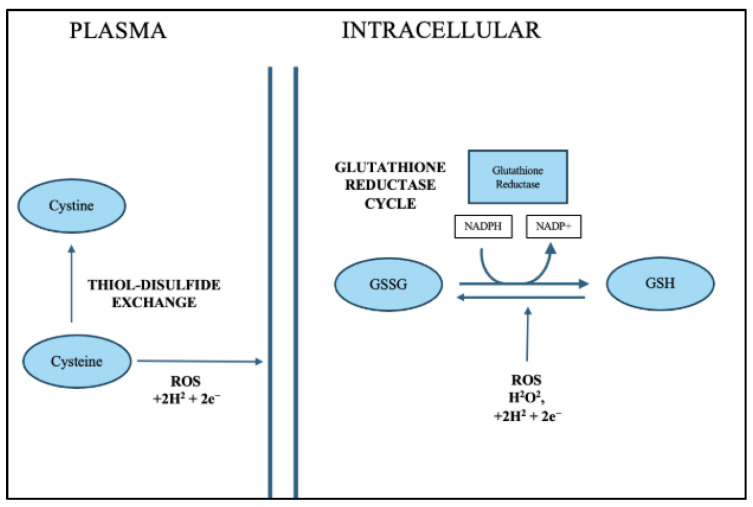
Schematic of Cysteine–Glutathione–ROS Redox Pathway under Normal Physiological Condition. Under normal conditions, plasma cysteine and intracellular glutathione cycles maintain a balanced thiol–disulfide state. Cysteine (CySH) and glutathione (GSH) exist predominantly in reduced forms, sustained by thiol–disulfide exchange reactions and glutathione reductase (GR) activity. Cystine from the plasma is taken up through the xCT transporter and reduced to cysteine inside the cell, supporting continuous GSH synthesis. GSH acts as the main antioxidant that neutralizes reactive oxygen species, or ROS, such as hydrogen peroxide. As GSH reduces ROS, it is converted to its oxidized form, GSSG. Glutathione reductase then uses NADPH to recycle GSSG back into GSH, maintaining the redox balance. The intracellular [GSH]/[GSSG] ratio exceeds 100:1, reflecting a highly reducing environment that supports redox signaling and protection against spontaneous oxidation. Together, these systems preserve a healthy intracellular environment by balancing oxidation and reduction reactions.

Calculated redox potentials (Eh) provide a quantifiable measure of the oxidative environment, integrating the proportional changes in reduced and oxidized thiols. The 5–10 mV shift toward more oxidized Eh values at 48 h are biologically meaningful, as even small changes in redox potential can alter enzyme activity, transcription factor regulation, protein folding, and cell survival pathways [21]. These findings support the potential utility of plasma thiol redox couples as non-invasive biomarkers capable of detecting evolving reperfusion injury when oxidative stress becomes systemically significant [21,22]. Maintenance of redox homeostasis is essential for normal cellular physiology, and the cysteine–glutathione system is critical in this process. Under normal conditions, the intracellular redox state is tightly controlled by the continuous interconversion between GSH and GSSG and the thiol–disulfide cycling of cysteine and cystine. (Figure 5). GSH acts as the principal low-molecular-weight antioxidant, donating electrons to neutralize ROS such as hydrogen peroxide (H_2_O_2_) and organic peroxides [23,24]. During this reaction, GSH is converted to its oxidized form, GSSG, which is subsequently reduced back to GSH by glutathione reductase using NADPH as a reducing equivalent. This cycle maintains a high intracellular GSH/GSSG ratio, often exceeding 100:1, thereby preserving a reducing intracellular environment [5].

The cysteine–cystine cycle functions in parallel to sustain intracellular cysteine pools required for GSH synthesis and redox regulation. Cystine, the oxidized dimer of cysteine, is imported into cells via the xCT (SLC7A11) antiporter in exchange for glutamate and subsequently reduced to cysteine by NADPH-dependent thioredoxin systems [25,26]. Cysteine not only serves as the rate-limiting substrate for glutathione biosynthesis but also participates in thiol–disulfide exchange reactions that maintain protein conformation and regulate signaling pathways sensitive to the redox state. Together, the interplay between the cystine/cysteine shuttle, glutathione turnover, and NADPH metabolism establishes a robust redox buffering system that allows cells to detoxify ROS and sustain metabolic balance under physiological conditions [27].

**Figure 6:**
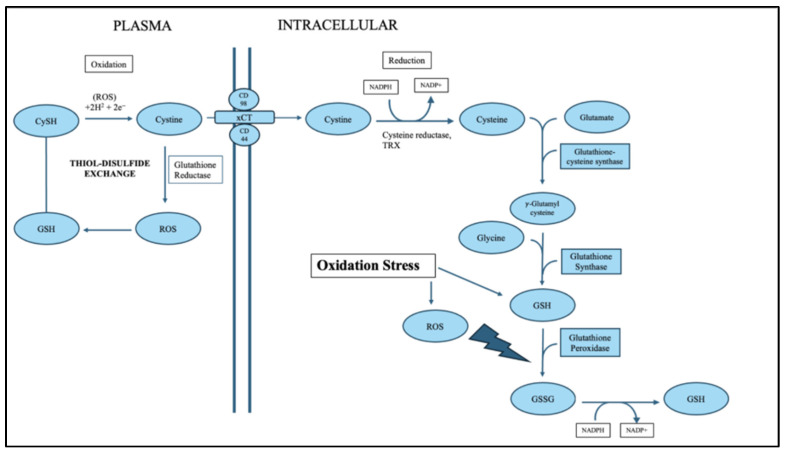
Schematic of Cysteine-Glutathione-ROS redox pathways under oxidative stress conditions in the plasma. This diagram illustrates how the cellular redox network responds to oxidative stress, emphasizing how glutathione metabolism, thiol-disulfide exchange, and amino acid synthesis interact to preserve redox balance when reactive oxygen species (ROS) accumulate. Under oxidative stress, intracellular levels of ROS such as hydrogen peroxide (H_2_O_2_), superoxide (O_2_•^−^), andhydroxyl radicals (•OH) rise dramatically. These ROS oxidize thiol groups in cysteine and glutathione molecules, leading to the formation of disulfide bonds (–S–S–) and disrupting normal cellular function. To counter this, cells activate a redox buffering system centered around glutathione (GSH), cysteine (CySH), and NADPH. The diagram shows the interconnected biochemical reactions that restore redox balance.

However, under oxidative stress conditions, this equilibrium becomes disrupted as ROS production exceeds the detoxifying capacity of the glutathione system (Figure 6). Mitochondrial respiration, xenobiotic metabolism, or inflammatory processes can elevate intracellular ROS, leading to oxidation of GSH and cysteine and accumulation of GSSG and cystine [28,29]. In response, antioxidant enzyme systems are upregulated to restore redox balance. Glutathione peroxidase (GPx) catalyzes the reduction in peroxides using GSH as an electron donor, while glutathione reductase (GR) regenerates GSH from GSSG using NADPH, thus sustaining redox cycling [5,24]. Additionally, the thioredoxin (TRX) and peroxiredoxin (PRX) systems complement the GSH cycle by reducing oxidized protein disulfides and peroxides, further expanding the cellular antioxidant capacity [30].

During oxidative stress, cells also activate de novo GSH synthesis to compensate for depletion. This synthesis proceeds via a two-step ATP-dependent process catalyzed by γ-glutamylcysteine synthetase (GCL) and glutathione synthase (GS), forming γ-glutamylcysteine and subsequently GSH from glutamate, cysteine, and glycine [5,31]. Cysteine availability becomes a key determinant of this process, linking sulfur amino acid metabolism directly to antioxidant defense. Methionine contributes indirectly through the transsulfuration pathway, replenishing cysteine pools when extracellular supply is limited [32]. Enhanced cystine import via xCT, coupled with cysteine reduction and GSH biosynthesis, constitutes an adaptive mechanism that protects cells from irreversible oxidative damage.

Furthermore, thiol/disulfide exchange reactions remain critical under stress conditions by modulating the activity of redox-sensitive proteins, transcription factors, and enzymes involved in cell survival and apoptosis. These reversible modifications serve as molecular switches that fine-tune signaling pathways such as NF-κB, Nrf2, and AP-1, ensuring appropriate cellular responses to oxidative stimuli [27,33]. Collectively, the cysteine–glutathione–ROS redox network represents an integrated system that senses and responds to oxidative fluctuations, coupling metabolism, redox buffering, and gene regulation to maintain cellular integrity.

The progression of oxidation observed here coincides with several known drivers of delayed ischemic injury, including the surge in mitochondrial ROS, release of damage-associated molecular patterns (DAMPs), activation of peroxiredoxin and thioredoxin pathways, and the onset of lipid peroxidation associated with ferroptosis. Iron-dependent oxidative mechanisms, depletion of antioxidant reserves, and enhanced inflammatory signaling likely interact synergistically, contributing to worsening oxidative imbalance at 48 h. These systemic redox changes may reflect processes occurring within the brain, suggesting that peripheral biomarkers could serve as proxies for cerebral biochemical status.

The exclusive use of male rats limits generalizability, as sex differences in redox biology, hormone-dependent neuroprotection, and inflammatory responses could alter redox trajectories. Future work will incorporate both sexes and evaluate whether redox biomarkers behave differently in female animals across the peri-ischemic period.

The study also focused on plasma biomarkers and did not include brain tissue redox measurements, which would strengthen mechanistic interpretations. Additional intermediate and late time points, analysis of antioxidant enzyme activity, and inclusion of female and aged animals would further clarify the redox response to ischemia–reperfusion.

Identifying the temporal window during which systemic oxidative stress becomes pronounced may inform therapeutic strategies targeting redox imbalance. Antioxidant interventions, redox-modulating agents, or glutathione-replenishing therapies may be more effective when administered during the delayed oxidative phase rather than immediately at reperfusion. The results also support the potential clinical value of plasma thiol redox measurements as biomarkers for monitoring secondary injury progression and treatment response following ischemic stroke.

## 5. Conclusions

This study demonstrates a clear, time-dependent progression of oxidative stress in plasma following tMCAO. Analysis of plasma thiol/disulfide concentrations, redox ratios, and calculated redox potentials (Eh) revealed that systemic redox homeostasis is initially maintained during early reperfusion (24 h) but undergoes a marked oxidative shift by 48 h. Together, these findings reflect the dynamic balance between antioxidant defenses and oxidative burden during cerebral ischemia. These findings align with prior reports that ischemia–reperfusion triggers ROS overproduction, mitochondrial dysfunction, and glutathione depletion. The late onset of redox imbalance suggests a delayed but progressive oxidative insult.

## Data Availability

The original contributions presented in this study are included in the article. Further inquiries can be directed to the corresponding authors.

## Abbreviations

The following abbreviations are used in this manuscript:

ROS	Reactive Oxygen Species
GSH	Glutathione
GSSG	Glutathione Disulfide
CySH	Cysteine
CySS	Cystine
tMCAO	Transient Middle Cerebral Artery Occlusion
CCA	Common Carotid Artery
ECA	External Carotid Artery
ICA	Internal Carotid Artery
MCA	Middle Cerebral Artery
Eh	Redox Potential
Ox	Oxidized
Red	Reduced
CBF	Cerebral Blood Flow
TTC	2,3,5-triphenyltetrazolium chloride
FJB	Flouro-Jade B
